# Single metachronous bone metastasis following rectal adenocarcinoma: A case report

**DOI:** 10.3892/mi.2024.130

**Published:** 2024-01-03

**Authors:** Belén Matías-García, Fernando Mendoza-Moreno, Manuel Díez-Alonso, Enrique Ovejero-Merino, Cristina Vera-Mansilla, Alma Blázquez-Martín, Ana Quiroga-Valcárcel, Rubén Jiménez-Martín, Rebeca D'amico, Inmaculada Lasa-Unzúe, Alberto Gutiérrez-Calvo

**Affiliations:** Department of General and Digestive Surgery, Príncipe of Asturias Teaching Hospital, Alcalá de Henares, 28805 Madrid, Spain

**Keywords:** colorectal cancer, bone metastases, survival, KRAS, liver, lung

## Abstract

Colorectal cancer (CRC) ranks as the third leading cause of cancer-related mortality in developed countries. While its incidence in early stages has increased due to screening programs, a significant number of patients experience the development of metastases either at the time of diagnosis or during follow-ups. Unlike certain other types of cancer, such as breast, prostate, or lung cancer, where bone tissue is a common site for secondary dissemination, CRC primarily spreads to the lymph nodes, liver and lungs. The occurrence of bone metastases from CRC is rare and usually coincides with tumor involvement in other locations. Risk factors for bone metastases include the location of the primary tumor, the age of the patients, KRAS mutations and the degree of tumor differentiation. Unlike metastases to the liver and lungs, bone metastases tend to be symptomatic, affecting the patient's quality of life and resulting in a poorer prognosis with shorter survival rates. The approach to patient management needs to be personalized. The present study describes the of a patient who underwent surgery for stage IV rectal adenocarcinoma and later developed a metastasis in the costal wall 79 months post-intervention, with no evidence of recurrence at other sites.

## Introduction

Colorectal cancer (CRC) ranks as the third leading cause of cancer-related mortality in developed regions, with ~1.2 million new cases diagnosed globally each year (1). Bone metastases constitute 6% of all CRC metastases (2). Notably, ~22% of patients exhibit distant metastases at the time of diagnosis, and 70% develop these during follow-up (3).

Despite their low incidence rate, bone metastases signify a worsened oncological prognosis. Metastasectomy may be a therapeutic option for singular and well-localized lesions; however, generally, treatment pursues a palliative goal. This involves systemic chemotherapy (with or without targeted therapy), radiotherapy, or the pharmacological use of bisphosphonates (4).

The present study describes the case of a patient who underwent surgery for stage IV rectal adenocarcinoma and developed a single bone metastasis at the costal level 79 months following the initial surgery. The description of this clinical case reminds us that despite a long follow-up period and radical surgery, the clinical monitoring of patients with metastatic rectal cancer should continue. On the other hand, the present study also describes the use of a bilaminar mesh (Ventralight ST Bard^®^) for repairing the defect in the chest wall following bone metastasectomy.

## Case report

A 61-year-old male patient with a history of dyslipidemia underwent surgery at the Príncipe of Asturias Teaching Hospital (Madrid, Spain) in October, 2013 for T3N0M1 rectal adenocarcinoma, which involved low anterior resection and liver metastasectomy from segment V. The pathological report indicated a poorly differentiated colorectal adenocarcinoma, with no lymph node involvement, perineural or lymphovascular invasion, or tumor deposits. The resection margins were free of tumor involvement. KRAS was determined to have a wild-type expression (data not shown). In relation to the metastasectomy, the colorectal origin was confirmed, with no involvement of the surgical margins.

**Figure 1:**
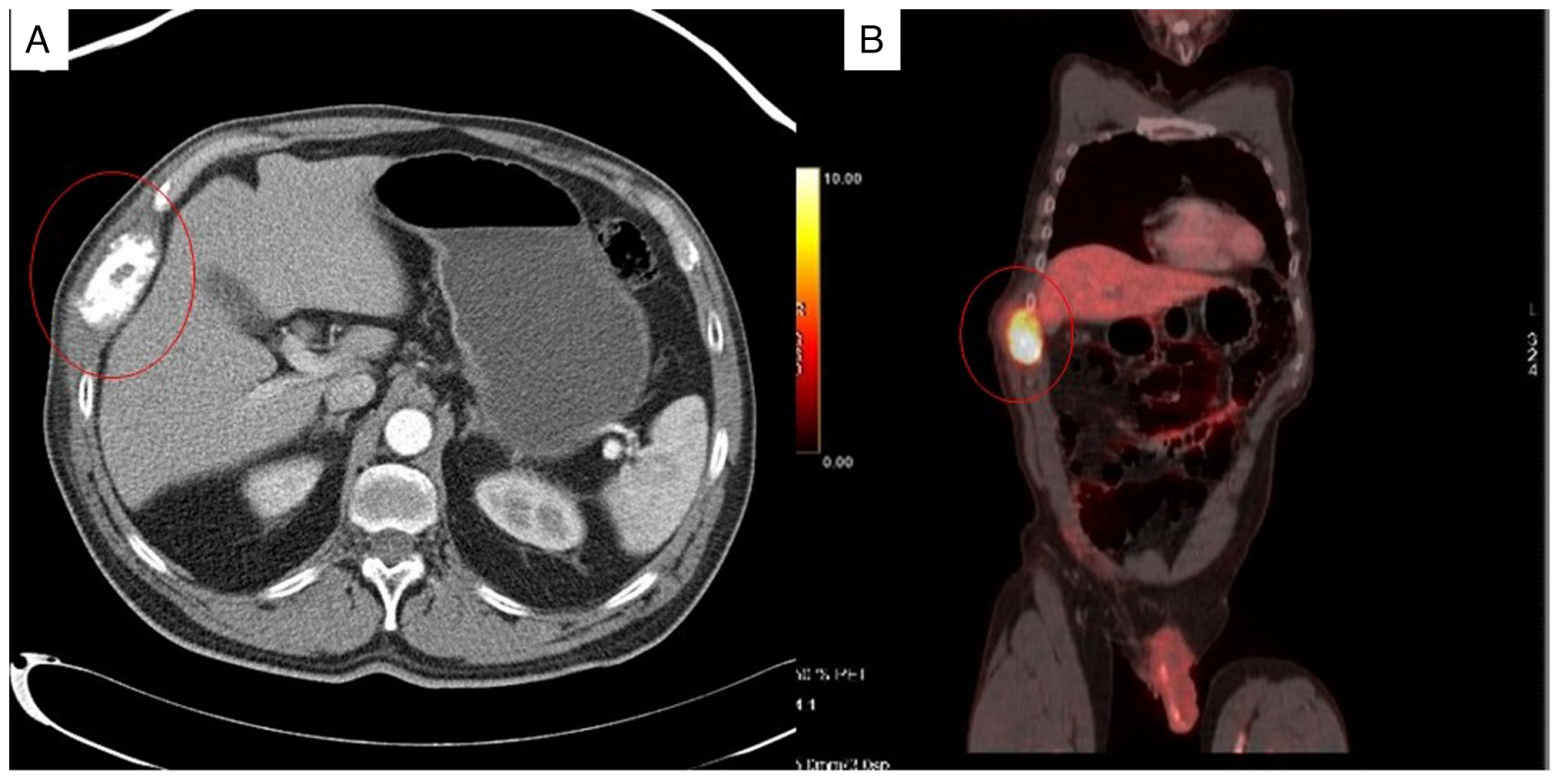
(A) Computed tomography scan illustrating bone metastases in the ninth right costal arch. (B) Positron emission tomography and computed tomography with hypermetabolic activity coincident with a mass described in the previous computed tomography scan (A) in the right costal region.

Adjuvant treatment included six cycles of the XELOX scheme (intravenous oxaliplatin 130 mg/m^2^ (day 1) followed by oral capecitabine 1,000 mg/m^2^ twice daily (day 1, evening, to day 15, morning). On May, 2020, the patient returned to Príncipe of Asturias Teaching Hospital reporting rib cage pain, which led to the discovery of a bone metastasis. An initial radiological study was performed consisting of a posteroanterior chest X-ray that revealed the alteration at the cortical level of the anterior arch of the right ninth rib in relation to lytic tumor. A complementary study was performed, using thoraco-abdominopelvic computed tomography and positron emission tomography and computed tomography, which revealed a hypermetabolic lesion at the level of the right chest wall with destruction of the arch of the ninth rib accompanied by a soft tissue mass measuring 69x44x67 mm with pathological uptake (SuvMax of 26.6) without other metabolically significant lesions (Fig. 1). Following this series of diagnostic tests, a biopsy confirmed colorectal adenocarcinoma. The patient received radiotherapy and the CAPOX regimen (130 mg/m^2^ oxaliplatin on day 1 and 2,000 mg/m^2^ oral capecitabine on days 1-14, every 3 weeks) with a subsequent positron emission tomography and computed tomography revealing no evidence of tumor progression. Following successful treatment, surgery was performed in May, 2021, involving the resection of the costal wall lesion and chest wall repair with a bilaminar mesh (Ventralight ST^®^, Bard).

On May 2020, he reported pain at the rib cage level in relation to the appearance of a soft tissue tumor. A biopsy was performed using core needle biopsy which revealed bone tissue infiltrated by an adenocarcinoma of colorectal origin, with a focal and weak expression for CK7 and an intense expression of CK20 and CDX2. Following the diagnosis, treatment with radiotherapy and a systemic treatment regimen was carried out according to the CAPOX scheme (capecitabine and oxaliplatin) (eight cycles) and subsequent re-evaluation using a new positron emission tomography and computed tomography scan that revealed no evidence of tumor progression.

An immunohistochemical analysis was also performed on the samples (thickness of 3 microns). The sections were embedded in paraffin for processing. The primary antibody dilution used was as follows: CK7 (factory pre-diluted; cat. no. GA619; clone D5/16B4; Agilent Technologies, Inc.; 23˚C, 12.5 min); CK20 (factory pre-diluted; cat. no. GA777; clone KS 20.8; Agilent Technologies, Inc.; 21˚C, 18 min); CDx2 (factory pre-diluted; cat. no. GA080; clone DAk-CDX2; Agilent Technologies, Inc.; 23˚C. 15 min). The secondary antibody dilution was performed by pre-dilution with biotin/streptavidin (cat. no. GV800; Agilent Technologies, Inc); also at 23˚C for 15 min. The counterstain used was hematoxylin/eosin (Abbey Color, Inc., with processing at 23˚C for 7 min). All samples were observed using a brightfield microscope (Nikon Eclipse 50i; Nikon Corporation) at x100 magnification

**Figure 2:**
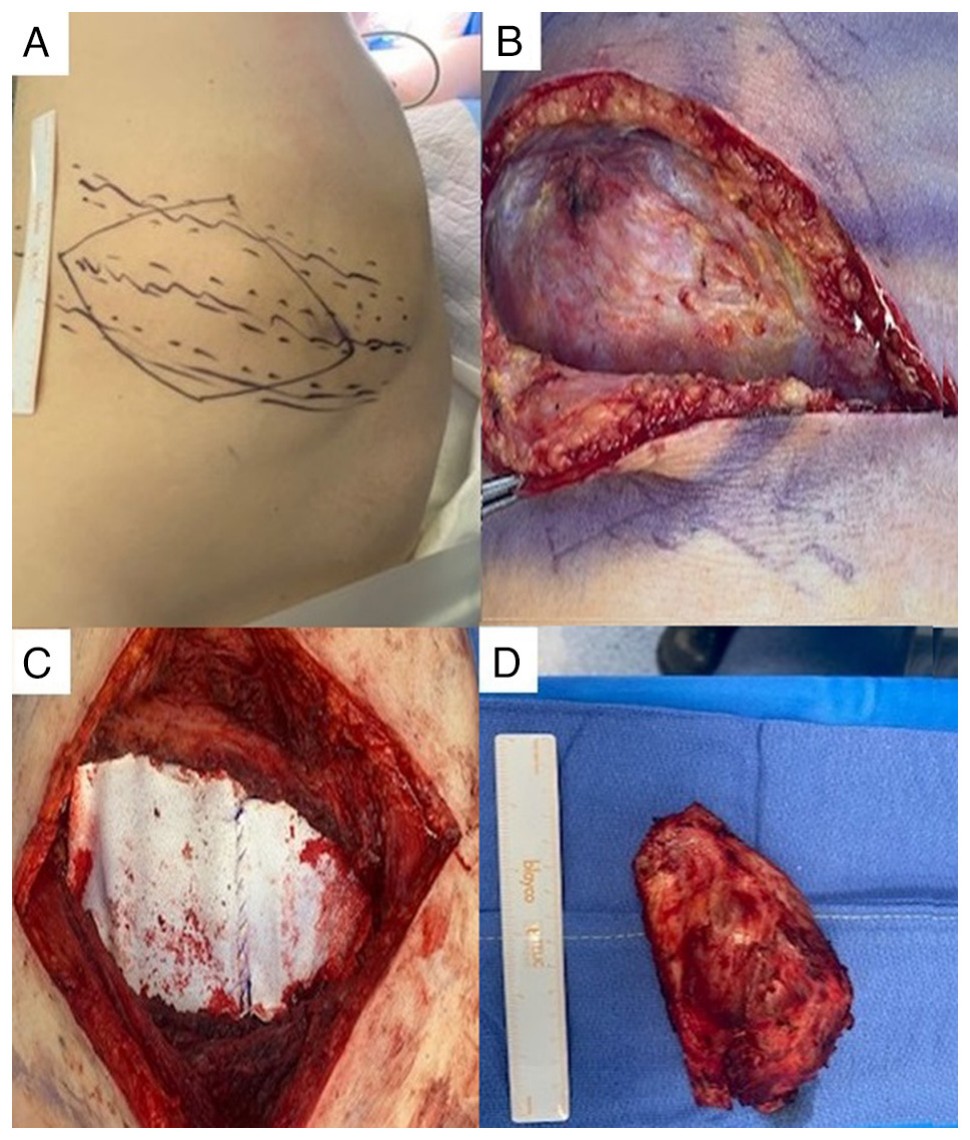
(A) Mass effect on costal region. (B) Exposure of tumor mass located in the eighth and ninth right costal arch. (C) Costal wall repair using bilaminar mesh. (D) Surgical section of bone metastasis removed.

Following the completion of the treatment and given the good response, surgery was performed in May, 2021, performing a right posterolateral thoracotomy. A tumor of the costal wall was observed involving the eighth and ninth right costal arch without diaphragmatic or pulmonary involvement. A complete resection of the lesion was performed *en bloc*, including the middle third of the eighth and ninth right ribs, repairing the chest wall using bilaminar mesh (Bard^®^) and placing an endothoracic tube. The patient was discharged on the fifth post-operative day (Fig. 2).

Following the intervention, clinical follow-up was maintained, presenting multiple pulmonary recurrence on a thoracic CT-scan in July 2022. Systemic treatment was initiated according to the CAPOX scheme with bevacizumab (6 cycles) with a good response, maintaining no evidence of recurrence until September, 2023.

Post-treatment follow-up revealed pulmonary recurrence in July, 2022, prompting systemic treatment with CAPOX and bevacizumab (7.5 mg/kg intravenous), resulting in no recurrence until the last follow-up in September, 2023.

## Discussion

CRC primarily metastasizes to the liver, but it can also spread to other parts of the body, including the lungs, peritoneum and, less commonly, the bones. Bone metastases represent 6% of all metastases from CRC (2). When CRC spreads to the bones, it can be challenging to treat and often indicates an advanced stage of the disease.

Bone tissue is a rigid, yet dynamic organ subject to continuous processes of formation and repair throughout life. Bone remodeling, predominantly a metabolic process in adults, determines the structure and function of the skeleton. Consequently, bone tissue integrity requires coordinated activity between cells responsible for bone formation (osteoblasts) and bone resorption (osteoclasts) (5). In the clinical case reported herein, it is noteworthy that metachronous metastatic involvement occurred at 79 months following the initial surgical intervention. This may be explained by a mechanism of hematogenous dissemination with localization of quiescent tumor cells at the bone level.

Bone is a frequent site of metastatic involvement in various solid organ cancers (breast, lung, thyroid, prostate or kidney cancer). The most frequent location is the pelvis (35.3%), followed by the lumbar spine (25%), thoracic vertebrae (21.6%), sacrum (20.6%) and ribs (14.7%) (1). Secondary bone involvement can be found in lung cancer in up to 40-50% or up to 60-70% in prostate cancer. However, bone as the location of metastases from gastrointestinal malignancies represents <10% of cases (6).

The incidence of bone metastases of colorectal origin is variable (0.96-11.1%), being more frequent in patients with a diagnosis of rectal cancer, likely influenced by its vascularization, explaining hematogenous dissemination as the most frequent mechanism (1). However, a higher incidence has been described in autopsies, ranging between 8.6 and 27% (7).

The presence of bone metastases, unlike the lung or liver location, is usually symptomatic, with local pain symptoms due to bone tissue destruction, causing irritation of the periosteum and/or nerve injury, potentially leading to pathological fractures (6). Therefore, unlike metastases located in the lungs or liver, bone metastases are diagnosed earlier.

In their study Zhenghong *et al* (1) described a greater association of bone metastases in patients with a poor degree of tumor differentiation, similar to the patient in the present study. Other factors related to the development of bone metastases are rectal origin, lymph node involvement and the presence of lung metastases (8).

In the patient in the present study, immunohistochemical analysis was key for the diagnosis, since the bone location and the time of appearance (at 79 months since the initial intervention) rendered colorectal origin unlikely.

Cytokeratin (CK)20 is an intermediate filament protein found in the cytoskeleton of epithelial cells. In tumor pathology, CK20 is a critical immunohistochemical marker frequently expressed in CRC. Its expression, along with other markers, can help confirm a colorectal origin for metastatic tumors when the primary site is unknown.

**Figure 3:**
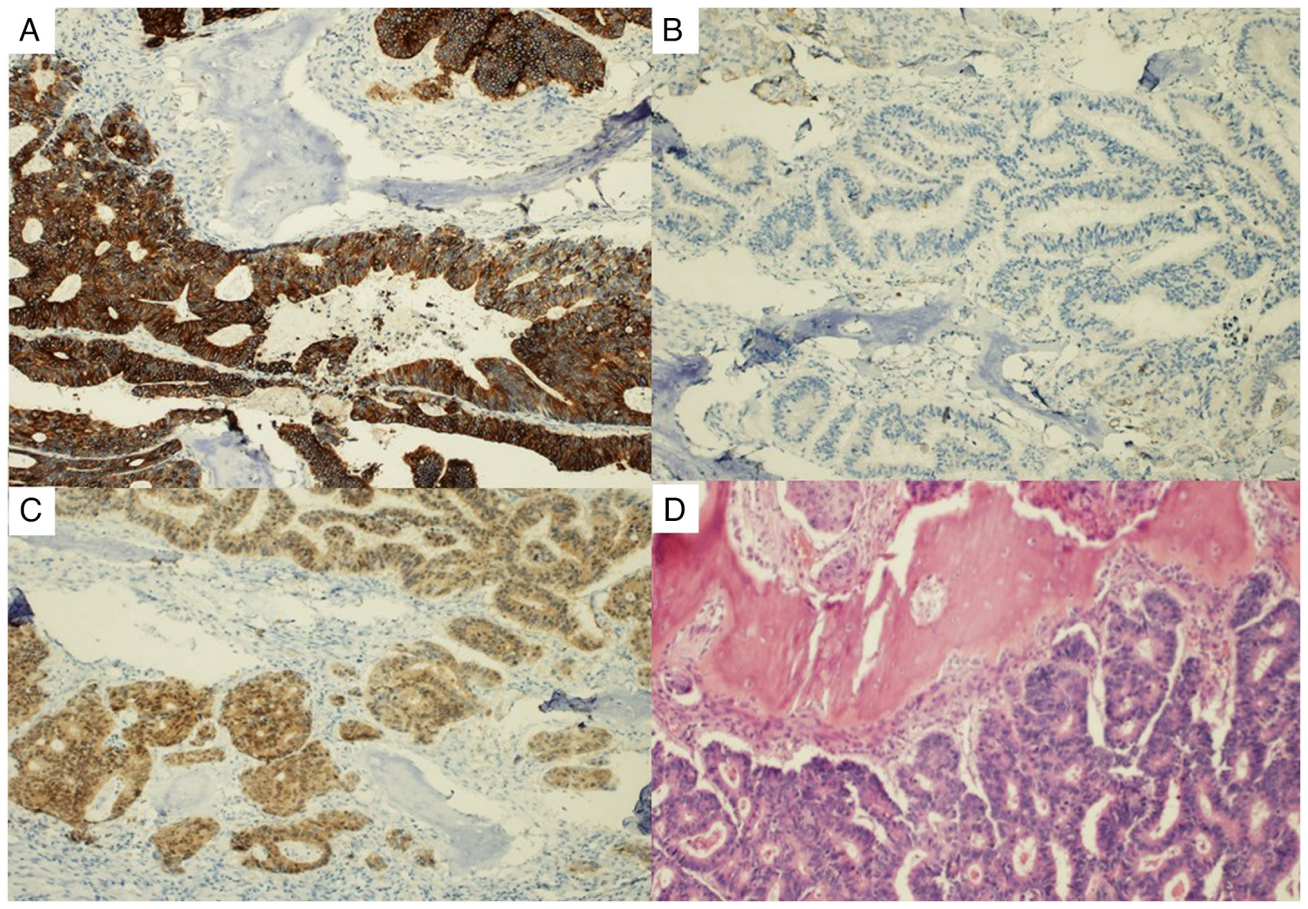
Metastasis colorectal adenocarcinoma involving the bone. (A) CDK20 diffuse positive. (B) CDK7 negative. (C) CDX2 diffuse positive. (D) Hematoxylin and eosin staining. Original magnification, x100.

The majority of primary lung adenocarcinomas are CK7-positive and CK20-negative (CK7^+^/CK20^-^). This CK7^+^/CK20^-^ pattern can help distinguish primary lung adenocarcinomas from metastatic colorectal adenocarcinomas, which are typically CK20^+^ and CK7(9). In the case described herein, the expression of CK7 was very weak and focal compared to CK20 and CDX2, which exhibited intense positivity (Fig. 3). In cases of metastatic carcinoma where the primary site is unknown, the expression pattern of CK7 and CK20 may be useful. For instance, if a metastatic tumor in the lung is CK20^+^/CK7^-^, one would consider potential primary sites, such as the colorectum. On the other hand, a CK20^-^/CK7^+^ pattern in a lung tumor would be more supportive of a primary lung origin (though other primary sites can also have this pattern).

The immunohistochemical profile of CRC is positive for CDK20 and CDX2, while it tends to be negative for CDK7. This association can be observed in Fig. 3, noting how in the bone tissue, the CDK20 marker is negative, whereas it exhibits positivity in the bone fragment infiltrated by the tumor. The same is true for CDX2 expression, negative in the bone tissue and positive in the colorectal tissue. CDX2 is a nuclear transcription factor primarily expressed in the cells of the intestinal epithelium, both in the small and large intestine. It plays a role in the development and differentiation of these cells. In the context of CRC, CDX2 is of interest as the loss of CDX2 expression in primary CRC has been shown to be associated with a worse prognosis (10). According to Cowan *et al* (11), this marker is expressed in ~12% of patients diagnosed with lung cancer, and its expression can be variable. In the context of prostatic carcinoma, the utility and prevalence of CDX2 expression are not widely recognized or documented (12).

The management of bone metastasis is an essential aspect of cancer care, particularly since the bone is a common site for metastasis for various primary tumors such as breast, prostate, lung, thyroid and kidney cancer (2). The effective treatment of bone metastasis can improve the quality of life of patients, and can decrease pain and reduce complications.

The treatment of bone metastases should include the administration of systemic chemotherapy and surgical resection when possible due to location. In the patient in the present study, given the absence of metastatic disease at other levels and the possibility of performing a resection with free surgical margins, this was performed. Treatment with radiotherapy was also performed with the aim of improving symptoms and reducing tumor size.

Previous studies have revealed that treatment with radiotherapy reduces bone pain and prevents pathological fractures, in addition to increasing survival in metachronous bone metastases (4). In the patient described herein, the aim of pre-operative radiotherapy was to reduce bone pain. Furthermore, the patient received six cycles of chemotherapy according to the CAPOX regimen with bevacizumab. According to the literature, the use of this type of systemic treatment regimen in patients with metastatic colorectal cancer is effective in terms of survival without compromising the quality of life of patients (13).

The 5-year survival rate of patients with lung or liver metastases is 8 and 16.9%, respectively. Some researchers have determined that, in the case of bone metastases, the 5-year survival is 3.4% (14). The presence of bone metastasis, together with age, location in the ascending colon, a high degree of differentiation, lymph node involvement and high pre-operative carcinoembryonic antigen levels have been described as poor prognostic factors in relation to the survival of patients undergoing surgery for CRC (15).

KRAS mutations occur in 40-50% of patients diagnosed with CRC (16). In previous studies, the authors described a negative association between patients with mutated KRAS with a diagnosis of CRC and the presence of peritoneal or liver metastases in terms of survival (17,18). In the patient in the present study, the KRAS determination corresponded to KRAS wild-type, providing a better prognosis and a better response to the administered systemic therapy.

In conclusion, CRC predominantly metastasizes to the liver and lungs, with bone metastasis being relatively rare. Its development is indicative of a poor prognosis. A comprehensive, multi-disciplinary approach is crucial in managing bone metastasis, considering the primary cancer type, stage and the performance status of the patient. The case presented herein emphasizes the importance of continued clinical monitoring and tailored treatment strategies for patients with metastatic CRC.

## Data Availability

The datasets used and/or analyzed during the current study are available from the corresponding author on reasonable request.
